# Can Vending Machines Promote Healthy Eating? Evidence from a Hospital Intervention

**DOI:** 10.3390/nu18020293

**Published:** 2026-01-16

**Authors:** Urška Rozman, Anja Kac, Miha Lavrič, Sonja Šostar Turk

**Affiliations:** 1 University of Maribor, Faculty of Health Sciences, Žitna ulica 15, 2000 Maribor, Slovenia; miha.lavric1@um.si (M.L.); sonja.sostar@um.si (S.Š.T.); 2 University of Maribor, Faculty of Agriculture and Life Sciences, Pivola 10, 2311 Hoče, Slovenia

**Keywords:** healthy vending, hospital, consumer behaviour, food environment, intervention study

## Abstract

Background/Objectives: Vending machines in hospitals offer convenient access to snacks and beverages for employees, visitors, and patients. However, their contents are typically energy-dense and nutritionally poor, which can potentially reinforce unhealthy eating habits. This study aimed to evaluate the impact of introducing healthier vending machine options on purchasing behaviour and consumer perceptions in a hospital setting. Methods: An interventional study was conducted at a university clinical centre in Slovenia. Sales data were collected from a standard vending machine and a pilot machine stocked with healthier products over two 14-day periods. Additionally, a consumer survey assessed factors influencing purchasing decisions and opinions on the healthier offerings. Results: The proportion of healthy items purchased increased from 22% to 39% in the pilot vending machine, indicating a positive shift toward healthier choices. However, total sales declined by 18.81%, suggesting consumer hesitation toward the new product mix. Survey results identified price, ingredients, and visual appeal as the primary factors influencing purchase decisions. Conclusions: The introduction of healthier vending machine options can promote better food choices in hospital environments, though challenges remain regarding consumer acceptance and sales performance. Expanding the variety of healthy items and adopting more competitive pricing strategies may enhance uptake. Further long-term research is needed to assess the sustainability of such interventions and their broader impact on hospital food environments.

## 1. Introduction

Vending machines offering food and beverages are a common feature of hospital environments and often represent the only readily accessible food source for employees, visitors, and patients seeking a quick meal or snack. This reliance became particularly pronounced during the COVID-19 pandemic, when hospital visiting restrictions further limited access to alternative food outlets, making vending machines primarily used by hospital staff. Healthcare workers, who frequently experience long and irregular working hours, often replace snacks or even main meals with products obtained from vending machines [1].

However, numerous studies indicate that vending machines in healthcare settings predominantly offer energy-dense, nutrient-poor products high in sugar, fat, and salt [2,3,4,5]. Such food environments may contribute to poor dietary quality and adverse health outcomes among healthcare workers, a population already shown to have elevated rates of unhealthy eating patterns and nutrition-related risk factors [6,7,8,9]. Improving the nutritional quality of food environments in hospitals is therefore a public health priority. Hospitals are increasingly recognised as settings that should model health-promoting environments; however, evidence consistently shows a mismatch between this role and the nutritional quality of foods available through vending machines [2,3,4,5]. This contradiction is particularly problematic given the reliance of healthcare workers on vending machines due to shift work, time pressure, and limited food access [6,7,8,9].

At the policy level, Slovenia’s National Assembly adopted the National Program on Nutrition and Health-Enhancing Physical Activity 2015–2025, coordinated by the Ministry of Health, which identifies nutrition at the workplace as a key priority area [10]. One of its specific goals is to improve the quality of food offerings and strengthen awareness of healthy eating among employees. In this context, hospitals represent a particularly relevant setting for interventions targeting workplace nutrition.

Environmental interventions that modify food availability, accessibility, and affordability—such as changes in vending machine offerings—have been proposed as effective strategies to promote healthier food choices at worksites. Previous studies conducted in hospital settings suggest that increasing the availability of healthier options in vending machines can positively influence purchasing behaviour [1,11,12,13,14,15,16,17]. Hospital-based intervention studies similarly report increases in healthier purchases following changes to vending machine offerings [18,19]. Many studies focus primarily on sales data [20,21,22,23,24,25,26,27] without incorporating consumers’ perceptions and acceptance of healthier vending options, while evidence from Central and Eastern European healthcare settings is scarce. Moreover, there is a lack of intervention studies that simultaneously assess behavioural outcomes and consumer attitudes following changes in vending machine offerings.

To address these gaps, the aim of this study was to evaluate the impact of modifying vending machine food and beverage offerings on purchasing behaviour and consumer perceptions in a hospital setting. The specific objectives were as follows:

(1) To assess changes in purchasing patterns following the introduction of healthier vending machine options;
(2) To evaluate consumers’ attitudes and satisfaction with the modified vending machine offerings;
(3) To explore the perceived acceptability of healthier vending machines among hospital users.

By providing empirical evidence from a hospital-based intervention in Slovenia, this study contributes to the growing literature on workplace food environment interventions and offers practical insights for policymakers and hospital administrators seeking to promote healthier eating through structural changes in food provision.

## 2. Materials and Methods

Study design: The present study was conducted at a university clinical centre in Slovenia, specifically at one of the selected clinics, in cooperation with the existing vendor of vending machines. Sales were monitored in two phases: the first 14 days on the existing classic vending machine with the sprout offer and the second 14 days on the pilot vending machine with the modified “healthier” offer. The study was conducted in October 2022, when some restrictive measures were still in effect due to the COVID-19 pandemic.

Intervention: The pilot vending machine with a “healthier” offer was filled according to recommendations for filling vending machines [28], considering specific goals of the National Program on Nutrition and Physical Activity for Health 2015–2025 (Ministry of Health of the Republic of Slovenia 2015). In the recommendations, the offer in vending machines is divided into two categories: a less healthy choice (20% of the offer) and a healthier choice (80% of the offer). The products that belong in a healthier choice category are precisely defined for each food category [29] based on their nutritional profile, energy density, and mass. Individual products within the category are limited by the maximum permitted contents of fat, saturated fatty acids, sugar, added sugar, and salt, which, according to the recommendations, represent 80% of the offer. The remaining 20% of food items include especially sweet drinks (beverages with added sugar), as well as all products with sweeteners, those that exceed the maximum permitted amount of salt, fat, and saturated fatty acids, and sandwiches with inappropriate fillings. The recommendations also list some exceptions that are allowed in the offer due to the high content of dietary fibre (>3 g/100 g of product) or suitability for people with special dietary needs (lactose intolerance, coeliac disease).

Data collection and analysis: Sales data were collected for ten hours a day, five days a week, by entering purchases using the MS Forms tool. Each of the sold products was categorised according to the Global Food Monitoring Group’s food categorisation system [29] and assigned to one of the 11 food categories, as shown in Table 1.

For each product, we obtained information on the weight/volume of the product, total fat, saturated fatty acids, total added sugar, and salt content per 100 g or 100 mL of the product. The obtained values were compared with the limit values set in the recommendations for filling vending machines [28] and thus each individual purchased product was labelled as a “healthy” or “less healthy” choice.

Each customer was invited to complete a survey consisting of six open-ended and closed questions. We aimed to analyse which products customers buy most often, the reasons for their purchase, whether they are missing some food items in the offer, how frequently, and in which establishments they usually make their purchases. At the pilot vending machine, we asked them about their opinions on the vending machine with healthy offerings in the hospital, what type of products they are most attracted to, the factors that are important to them when making a purchase, and whether they miss any products. Demographic questions regarding gender, age, and employment/student status were also collected. The data obtained were analysed using MS Excel.

Statistical Analysis: Descriptive statistics were used to summarise total sales volumes and the distribution of healthy and less healthy products across vending machine types.

To assess whether overall sales volume differed between the standard vending machine and the pilot vending machine during equivalent fourteen-day observation periods, an exact one-sided binomial test was applied. The null hypothesis assumed that the proportion of items sold from the pilot vending machine was equal to 0.5, corresponding to equal total sales between the two vending machine phases. The alternative hypothesis tested whether the proportion of items sold from the pilot vending machine was lower than 0.5.

To evaluate differences in purchasing behaviour, a 2 × 2 contingency table was constructed comparing healthy versus less healthy product purchases in the standard and pilot vending machines. Group differences were assessed using Fisher’s exact test, which was selected due to its robustness for categorical data and to avoid reliance on asymptotic assumptions. Effect size was quantified using odds ratios (ORs) with corresponding 95% confidence intervals (CIs). Statistical significance was set at *p* < 0.05. All analysis was performed using standard statistical procedures.

## 3. Results

**Figure 1:**
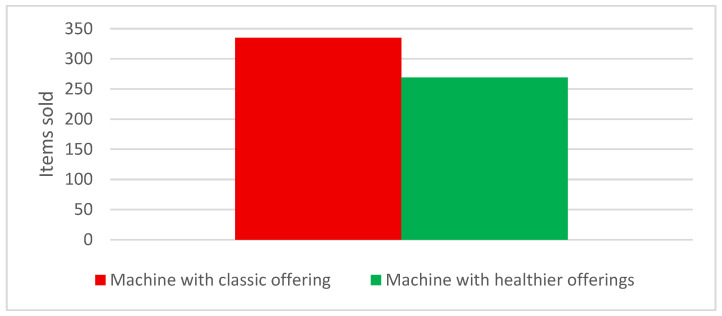
Total sales after one month on vending machines with classic or healthier offerings.

**Figure 2:**
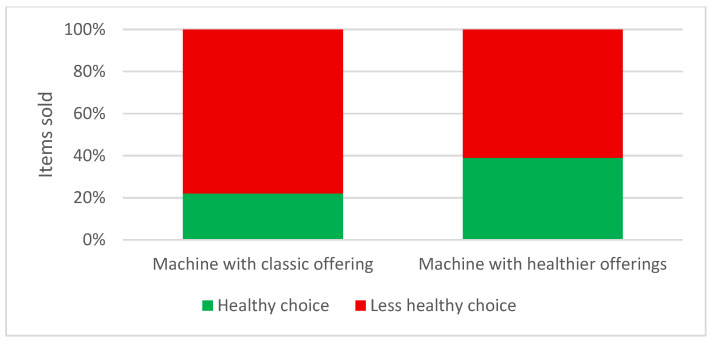
Percentage of healthy and less healthy choice items bought from vending machines with classic or healthier offerings.

Over the course of one month, a total of 604 items were sold, with 335 items sold on a classic vending machine and 269 items on a pilot vending machine, resulting in an 18.81% decline in overall sales (Figure 1). Of the items sold on both vending machines, an increase in buying healthy options was observed on the pilot vending machine, with 39% (*n* = 105) of total items bought being “healthy choice” versus 22% (*n* = 74) on the machine with classic offerings. Conversely, there was a decrease in buying less healthy choice items, with 61% (*n* = 164) of total items bought being “less healthy choice” on the pilot vending machine versus 78% (*n* = 261) “less healthy choice” of total items being purchased on the machine with classic offerings (Figure 2).

Over the 14-day period, 269 of 604 items (44.5%) were sold from the pilot vending machine. The exact one-sided binomial test showed that this proportion was significantly lower than the expected proportion of 50% (*p* ≈ 0.004), indicating a statistically significant reduction in total sales during the pilot phase.

A marked difference in purchasing patterns was observed between vending machine types. In the pilot vending machine, 105 of 269 items (39.0%) of the purchased items were classified as healthy, compared with 74 of 335 items (22.1%) in the standard vending machine. Fisher’s exact test demonstrated that this difference was statistically significant (*p* < 0.001). Customers were more than twice as likely to purchase a healthy product from the pilot vending machine than from the standard vending machine (OR = 2.26; 95% CI: 1.60–3.19), indicating a substantial shift toward healthier choices when healthier options were made more available.

The number of items sold per food group in each vending machine was recorded, with sweet drinks remaining the most sought-after items in both machines.

**Figure 3:**
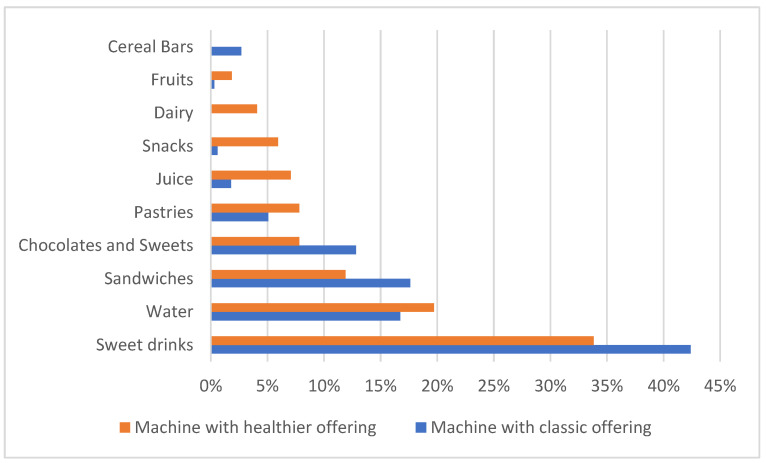
Visual comparison of the percentage of items sold per food group in the classic and the pilot vending machines.

When introducing vending machines with healthier offerings, there was an increase in water, juice, pastries, healthy snacks, and fruit consumption, as well as a decrease in the consumption of sweet drinks, sandwiches, and chocolate and sweets. Moreover, no dairy products were purchased and/or available in the classic vending machine, and no cereal bars were purchased and/or available in the pilot vending machine (Figure 3).

### 3.1. Survey Results at the Classic Vending Machine

**Figure 4:**
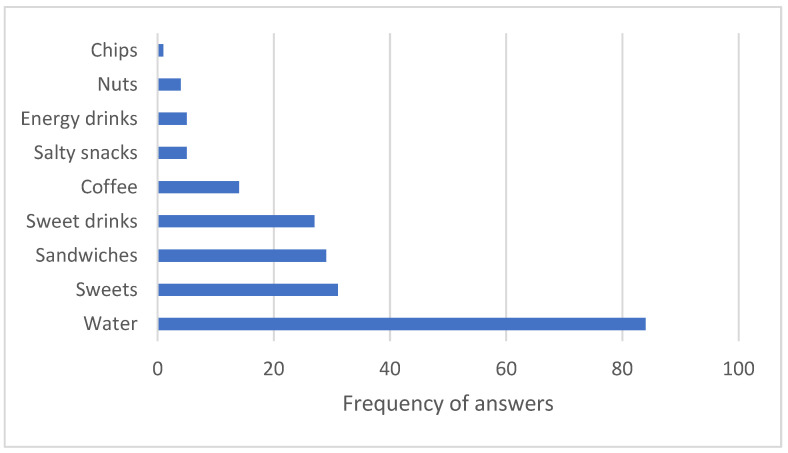
Reported numbers by survey respondents on which products they buy most often at vending machines.

**Figure 5:**
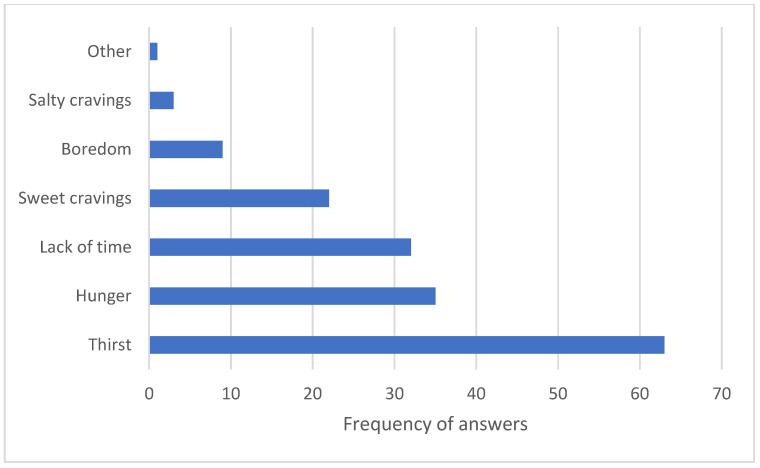
Reported numbers by survey respondents on the main reasons for purchasing products at vending machines.

In the survey performed at the classic vending machine, respondents reported which products they buy most often, with water being the most sought-after product (Figure 4). Interestingly, this does not correlate with the purchase numbers, as sweet drinks remain the most sought-after item. However, the main reason for buying vending machine items was thirst, as reported by survey respondents (Figure 5).

Survey respondents at the classic vending machine were mostly satisfied (86 satisfied vs. 38 not satisfied) with the selection of vending machine offerings. The types of foods that respondents would like to see in vending machines are very diverse. Many respondents indicated that they would like to see chips in vending machines. Some would like to see products with high protein content, rum bars, chewing gum, menthol candies, beer, yoghurt, a wider selection of sandwiches, and whole foods. Some respondents also expressed a desire for more fruits and products containing more vegetables.

**Figure 6:**
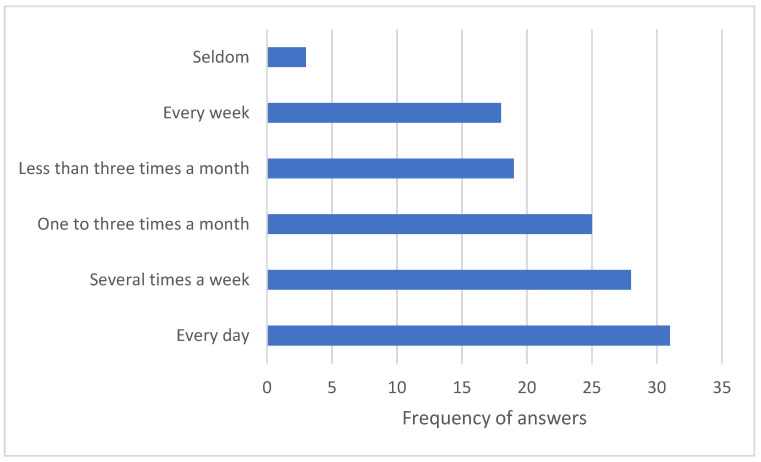
Purchase frequency at vending machines reported by survey respondents.

Concerning purchase frequency at vending machines, a total of 31 respondents answered that they purchase from vending machines every day, 28 respondents said they purchase from vending machines several times a week, 18 purchase every week, 25 purchase one to three times a month, and 19 purchase less than three times a month. Three people responded that they seldom shop at vending machines (Figure 6).

### 3.2. Survey Results at the Pilot Vending Machine

In the pilot vending machine with a modified food offering in the hospital, 226 customers were recorded over the fourteen-day study period. Out of these, 67 customers were surveyed. First, we asked the participants which vending machine they believe has more appropriate food or products for sale in the hospital or healthcare institution. Out of 67 participants, 48 respondents answered that, in their opinion, the vending machine with a healthier food or product selection is more appropriate. Only nineteen respondents believed that the vending machine with a standard food or product selection is more suitable for a hospital or healthcare institution.

**Figure 7:**
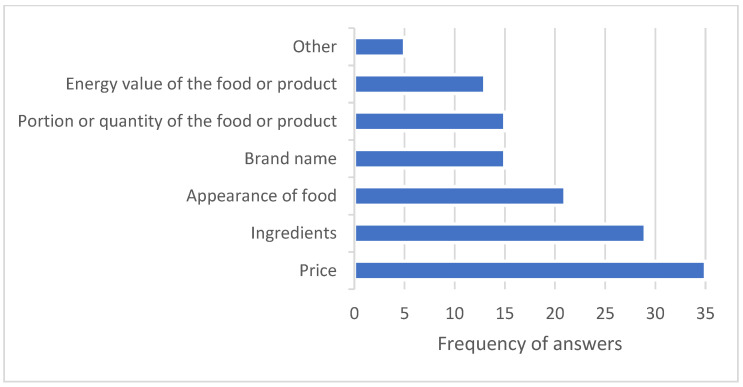
Factors that respondents consider important when deciding to purchase from a vending machine—a pilot vending machine in the hospital.

In the next survey question, we asked the participants about the factors they consider important when deciding to purchase from a vending machine (Figure 7). In this question, respondents were given the option to select multiple answers and could also specify any other factors they deemed important when purchasing food items.

Respondents mentioned as many as 36 times that the most important factor when purchasing is the price. Ingredients were selected as a factor in vending machine purchases 29 times, the appearance of the food 21 times, and the portion or quantity of the food or product 16 times. Respondents mentioned that the brand is important to them 15 times, and 13 times they mentioned the energy value of the food or product. Five respondents indicated other factors that influenced their choice. Some buyers mentioned time available for selection and physiological needs, such as hunger and thirst, as factors.

**Figure 8:**
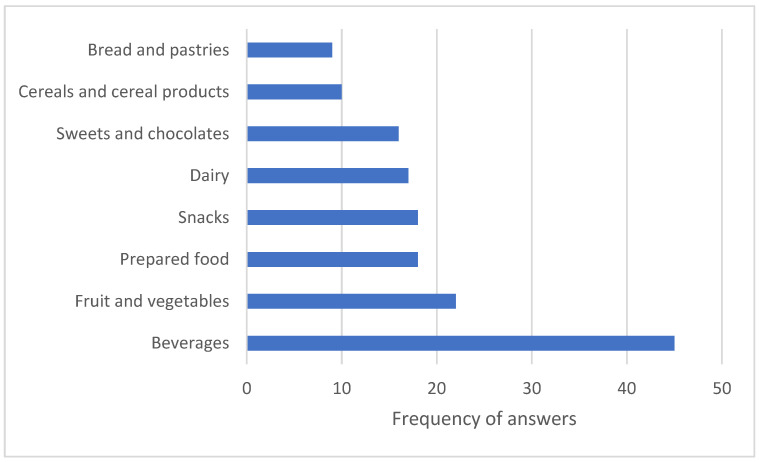
Types of products that attract respondents to the vending machine—a pilot vending machine in the hospital.

We were further interested in which type of products most attract respondents to a vending machine with a healthier choice (Figure 8).

In this survey question, we also offered respondents the possibility to choose multiple answers. The vast majority of respondents stated that they are most attracted to the category of beverages, which includes mineral water, juice, sugar-free or energy-free drinks, and other beverages. The category of fruit and vegetables followed, selected 22 times. Next were the categories of snacks and prepared food, each of which was selected 18 times. Dairy products were chosen 17 times, sweets 16 times, cereals and cereal products 10 times, and bread and pastries nine times.

The survey revealed that as many as 42 people in the future would, due to healthier food offerings, decide to purchase from vending machines more frequently. Of the 67 respondents, 6 indicated that they would not purchase more often due to the changed offerings, and 19 people did not care about the offer.

In this survey, we also asked participants if there was any food item they missed in the current vending machine offerings. Out of all participants, 45 people answered that they did not miss anything, while 22 people indicated that they would like to see a greater selection of sandwiches, such as a tuna sandwich, more yoghurts, and smoothies. Participants also mentioned that they would like to see fresh fruit, croissants, decaffeinated coffee, more non-carbonated beverages, a wider selection for children, and products suitable for vegetarians. One person also expressed a desire for more foods with low carbohydrate content and high fat content.

The results of the survey confirm the findings of French et al. [26], who stated that nutritional strategies have positive effects on consumer purchasing habits, although these effects are small in scale and short-lived. The results also align with the findings of Bos et al. [30], which note that the current offerings in vending machines contradict the public perception that healthcare institutions should promote healthy eating.

## 4. Discussion

The results of this intervention study highlight several important aspects regarding the impact of healthier vending machine offerings in a hospital setting. Despite an overall decrease in the total number of items sold, there was a noticeable shift toward healthier purchasing behaviour when healthier options were introduced. Specifically, the percentage of healthy products sold increased from 22% to 39%, indicating that providing healthier choices can indeed positively influence consumer decisions, even when unhealthy options are still available. This supports previous findings that consumers may opt for healthier choices when they are made more available and prominent [12,18]. Beyond product availability, complementary strategies such as nutrition labelling, visual cues, and promotional messaging have also been shown to enhance the effectiveness of healthy vending interventions. Recent studies indicate that point-of-sale communication strategies, including traffic-light labels, health claims, and visual nudges, can further influence consumer choices by increasing the salience and perceived attractiveness of healthier products. Field trials in vending machine settings have demonstrated that such labelling and messaging strategies can shift purchasing patterns toward healthier beverages and snacks, particularly when combined with improved product availability [27,31,32,33,34]. Boelsen-Robinson et al. [35] also reported that people increasingly expect healthcare institutions to set an example in terms of healthy eating.

However, the overall reduction in sales, with a decline of 18.81% in total items sold, suggests that while healthier options may be chosen more frequently, they do not necessarily drive higher consumption overall. This finding could reflect consumer hesitancy or resistance to change when presented with modified offerings, particularly in environments like hospitals where convenience and speed are often prioritised over nutrition [1]. But according to the findings of French et al. [25], lowering the price of healthy foods in vending machines could lead to increased consumption. Food choices in vending machine settings are shaped by a combination of behavioural, economic, and environmental determinants. Environmental factors such as product availability, visibility, and placement influence choice by shaping the default options available to consumers [36]. Economic determinants, particularly price sensitivity, play a central role, with multiple studies demonstrating that lower prices for healthier items are associated with increased purchasing [20,22]. Behavioural factors, including habits, taste preferences, and time constraints, further mediate purchasing decisions, especially among healthcare workers operating under high workload and stress [1,6]. These determinants align with socio-ecological and food environment models, which emphasise that individual food choices are strongly influenced by structural conditions rather than personal motivation alone. Moreover, previous vending machine interventions suggest that an initial decline in sales may reflect a temporary adaptation period, during which consumers adjust their expectations and preferences to a newly introduced product mix, with purchasing patterns stabilising over time [25,35]. Although our research found that total sales decreased significantly, the proportion of healthy purchases increased substantially, indicating a shift in purchasing behaviour rather than a simple reduction in demand.

An interesting contradiction was observed between reported preferences and actual purchases. While survey respondents indicated that water was the most commonly purchased item, the sales data showed that sweet drinks remained the most frequently purchased product across both vending machines. This mismatch suggests that while individuals may express a preference for healthier options such as water, their behaviour does not always align with these stated preferences, perhaps due to habit, taste preference, or ease of access to less healthy choices [4].

Another significant finding was the increased demand for healthier products, such as juices, pastries, and healthy snacks, as indicated by the percentage increase in their consumption from the pilot vending machine. This aligns with consumer interest in greater variety in healthier offerings, as evidenced by their requests for more sandwiches, yoghurt, and fruits, also confirmed by the findings of Rozman et al. [37]. Despite the success of the intervention in promoting healthier choices, a clear need remains to diversify the range of healthy products offered. The absence of items like cereal bars in the pilot machine likely contributed to fewer sales [25].

Price emerged as the most critical factor influencing purchase decisions, aligning with existing research that suggests affordability significantly affects the consumption of healthier options in vending machines [25]. Therefore, while making healthier choices more available, reducing their prices could further encourage healthier purchasing habits.

Evidence from intervention studies and reviews suggests that modifying hospital vending machine environments can lead to improvements in purchasing behaviour; however, findings remain context-dependent. Utter and McCray [1] note that policy directives alone are often insufficient to achieve meaningful changes, highlighting the importance of implementation strategies, consumer acceptance, and organisational support. More recent intervention studies further suggest that environmental modifications can influence purchasing behaviour, yet many evaluations focus primarily on sales outcomes and provide limited insight into consumer perceptions and acceptability [38].

Overall, this study reinforces the idea that environmental interventions, such as modifying vending machine offerings, can influence consumer behaviour towards healthier eating. However, it also highlights the importance of considering other factors like product diversity, pricing strategies, and consumer education to maximise the effectiveness of such interventions. Future research could investigate the longer-term effects of sustained changes to vending machines and test various pricing strategies to encourage even wider adoption of healthier options.

### Limitations

This study has several limitations that should be considered when interpreting the findings. First, the intervention was conducted at a single hospital site and involved only one vending machine location, which limits the external validity and generalisability of the results. Purchasing behaviour may vary across hospitals, departments, regions, and healthcare systems, depending on organisational culture, staff composition, and local food environments.

Second, the short duration of the intervention—two consecutive 14-day phases—limits the ability to assess longer-term behavioural adaptation. Changes in food purchasing behaviour may require extended exposure periods, particularly in institutional settings where habits and routines are well established. The observed trends, therefore, reflect short-term responses rather than sustained behavioural change.

Third, the study was conducted during a period in which COVID-19-related organisational constraints were still present. These circumstances may have influenced hospital staffing patterns, visitor numbers, and overall demand for vending machine products, thereby affecting both sales volume and purchasing decisions.

Fourth, sales data were recorded manually, which introduces a potential risk of recording error and limits the precision of the dataset. Although a structured data entry procedure was used, automated sales extraction would have provided greater reliability and granularity. Additionally, information on vending machine location, user flow, and visibility could not be systematically controlled and may have influenced purchasing behaviour.

Fifth, the analysis was descriptive in nature, and no inferential statistical testing was performed. As a result, the study does not allow conclusions regarding statistical significance or causal effects of the intervention. The findings should therefore be interpreted as indicative trends rather than definitive evidence of behavioural change.

Finally, although a consumer survey was conducted, self-reported preferences did not always align with observed purchasing behaviour, highlighting the complexity of food choice decision-making. The survey instrument was not based on a validated behavioural framework, and qualitative insights were limited by the brief format of the questionnaire.

Despite these limitations, the study provides valuable exploratory evidence on the feasibility and potential impact of modifying vending machine offerings in a hospital setting. It highlights key practical challenges—such as pricing, product mix, and consumer acceptance—that can inform the design of future, larger-scale and longer-term interventions across multiple healthcare institutions.

## 5. Conclusions

This study demonstrates the potential for vending machines to serve as an effective tool for promoting healthier eating habits in hospital settings. By modifying the food and beverage offerings to include a greater proportion of healthier options, a notable shift in consumer purchasing behaviour occurred, with an increase in the selection of healthier items. This suggests that the availability and accessibility of healthy options can influence consumer decisions. However, the overall decline in sales highlights some challenges, such as the need for greater variety and possibly lower prices for healthier items to encourage consistent purchasing. Additionally, the discrepancy between survey responses and actual purchasing behaviour points to the complexity of consumer preferences and behaviours, where stated health-conscious intentions may not always translate into action. Ultimately, this intervention supports the notion that environmental changes, such as healthier vending machine offerings, can promote better eating habits; however, further refinements, including product diversity and pricing strategies, are necessary to enhance their impact. Future studies could focus on long-term outcomes and explore strategies such as price adjustments and educational campaigns to further enhance the effectiveness of these interventions in promoting healthier dietary choices among hospital staff and visitors.

## Figures and Tables

**Table 1:** Foods categorisation.

(Sub) Category	Description/Examples
Juices	100% natural juice, smoothie
Soft drinks	carbonated beverages with sugar, carbonated drinks with no sugar and added aspartame/acesulfame-k sweetener, non-alcoholic beer, non-carbonated beverages with sugar, sugar-tasting water, water with taste but no sugar
Energy drinks	energy drinks with no sugar and added aspartame/acesulfame-k sweetener, energy drinks with sugar
Waters	mineral water, water
Coffee	iced coffee
Biscuits	biscuits, cookies, croissants, wafer
Cereal Bars	cereal tiles
Chewing Gum	chewing gum
Chocolate and Sweets	candy, chocolate, chocolate/chocolate snacks, fruit tiles
Crisps and Snacks	chips, crackers, flips, salted sticks/salty pretzels
Fruit	dried fruits, fresh fruits
Ice Cream and Edible Ices	ice cream
Milk	milk
Nuts and Seeds	nuts
Pre-prepared Salads and Sandwiches	fried cheese/chicken, prosciutto or ham, salami and sausages, tuna, vegetarian
Yoghurt Products	fruit, natural

## Data Availability

Data is contained within the article.
